# The Role of Artificial Intelligence in the Diagnosis and Treatment of Ulcerative Colitis

**DOI:** 10.3390/diagnostics14101004

**Published:** 2024-05-13

**Authors:** Petar Uchikov, Usman Khalid, Nikola Vankov, Maria Kraeva, Krasimir Kraev, Bozhidar Hristov, Milena Sandeva, Snezhanka Dragusheva, Dzhevdet Chakarov, Petko Petrov, Bistra Dobreva-Yatseva, Ivan Novakov

**Affiliations:** 1 Department of Special Surgery, Faculty of Medicine, Medical University of Plovdiv, 4000 Plovdiv, Bulgaria; puchikov@yahoo.com (P.U.); inovakov2003@yahoo.com (I.N.); 2 Faculty of Medicine, Medical University of Plovdiv, 4000 Plovdiv, Bulgaria; usmankhalid957@gmail.com; 3 University Multiprofile Hospital for Active Treatment “Saint George”, 4000 Plovdiv, Bulgaria; nikola.vankov@mail.bg; 4 Department of Otorhynolaryngology, Medical Faculty, Medical University of Plovdiv, 4000 Plovdiv, Bulgaria; kraevamaria93@gmail.com; 5 Department of Propedeutics of Internal Diseases, Medical Faculty, Medical University of Plovdiv, 4000 Plovdiv, Bulgaria; 6 Section “Gastroenterology”, Second Department of Internal Diseases, Medical Faculty, Medical University of Plovdiv, 4000 Plovdiv, Bulgaria; hristov.bozhidar@abv.bg; 7 Department of Midwifery, Faculty of Public Health, Medical University of Plovdiv, 4000 Plovdiv, Bulgaria; sandewa@abv.bg; 8 Department of Nursing Care, Faculty of Public Health, Medical University of Plovdiv, 4000 Plovdiv, Bulgaria; sdragusheva68@gmail.com; 9 Department of Anesthesiology, Emergency and Intensive Care Medicine, Medical Faculty, Medical University of Plovdiv, 4000 Plovdiv, Bulgaria; 10 Department of Propaedeutics of Surgical Diseases, Section of General Surgery, Faculty of Medicine, Medical University of Plovdiv, 4002 Plovdiv, Bulgaria; dchakarov@mail.bg; 11 Department of Maxillofacial Surgery, Faculty of Dental Medicine, Medical University of Plovdiv, 4000 Plovdiv, Bulgaria; petkogpetrov0@gmail.com; 12 Section “Cardiology”, First Department of Internal Diseases, Medical Faculty, Medical University of Plovdiv, 4000 Plovdiv, Bulgaria; bistra.yatseva@mu-plovdiv.bg

**Keywords:** artificial intelligence, ulcerative colitis, diagnosis, inflammatory bowel disease, artificial neural networks

## Abstract

Background and objectives: This review aims to delve into the role of artificial intelligence in medicine. Ulcerative colitis (UC) is a chronic, inflammatory bowel disease (IBD) characterized by superficial mucosal inflammation, rectal bleeding, diarrhoea and abdominal pain. By identifying the challenges inherent in UC diagnosis, we seek to highlight the potential impact of artificial intelligence on enhancing both diagnosis and treatment methodologies for this condition. Method: A targeted, non-systematic review of literature relating to ulcerative colitis was undertaken. The PubMed and Scopus databases were searched to categorize a well-rounded understanding of the field of artificial intelligence and its developing role in the diagnosis and treatment of ulcerative colitis. Articles that were thought to be relevant were included. This paper only included articles published in English. Results: Artificial intelligence (AI) refers to computer algorithms capable of learning, problem solving and decision-making. Throughout our review, we highlighted the role and importance of artificial intelligence in modern medicine, emphasizing its role in diagnosis through AI-assisted endoscopies and histology analysis and its enhancements in the treatment of ulcerative colitis. Despite these advances, AI is still hindered due to its current lack of adaptability to real-world scenarios and its difficulty in widespread data availability, which hinders the growth of AI-led data analysis. Conclusions: When considering the potential of artificial intelligence, its ability to enhance patient care from a diagnostic and therapeutic perspective shows signs of promise. For the true utilization of artificial intelligence, some roadblocks must be addressed. The datasets available to AI may not truly reflect the real-world, which would prevent its impact in all clinical scenarios when dealing with a spectrum of patients with different backgrounds and presenting factors. Considering this, the shift in medical diagnostics and therapeutics is coinciding with evolving technology. With a continuous advancement in artificial intelligence programming and a perpetual surge in patient datasets, these networks can be further enhanced and supplemented with a greater cohort, enabling better outcomes and prediction models for the future of modern medicine.

## 1. Introduction

Ulcerative colitis and Crohn’s disease are the primary compartments of inflammatory bowel disease (IBD), characterized by superficial mucosal inflammation, rectal bleeding, diarrhoea, and abdominal pain. The overlapping nature between the two conditions can cause confusion; however, distinguishing factors, including risk factors, spectrums of variability relating to genetic predisposition and histologic and endoscopic features aid physicians from a diagnostic standpoint. Inflammatory Bowel Disease aetiology has not been discovered, but advancing research suggests that individuals with a genetic predisposition exhibit an imbalanced mucosal immune reaction to the normal bacteria residing in the gut, which acts as a contributing factor in bowel inflammation [1,2]. Endoscopy, alongside histological evaluation, assumes a paramount role in the diagnosis and management of Inflammatory Bowel Disease (IBD), while also serving as a pivotal component in the surveillance of colorectal cancer [3]. The introduction of artificial intelligence (AI) would provide a new outlook in medical diagnostics and treatments, boosting patient care in a spectrum of varied clinical settings. Artificial Intelligence (AI) possesses the capacity to analyze vast quantities of intricate data at a markedly accelerated pace compared to human capabilities. It adeptly illuminates nuances that may elude our eyes, thus allowing a meticulous and unbiased assessment of the data at hand [4]. As a result of the vital application of endoscopic techniques and gastroenterological imaging modalities, especially in IBD, AI-based image analysis has the potential to be manipulated and incorporated in an array of situations covering a spectrum from appraising endoscopic lesions to detecting cancer and gauging disease activity and severity, including prognosis and treatment response assessment [3]. This review aims to delve into the role of artificial intelligence in medicine. By identifying the challenges inherent in UC diagnosis, we seek to highlight the potential impact of artificial intelligence on enhancing both diagnosis and treatment methodologies for this condition. AI aids in detecting mucosal lesions and automates the assessment of UC biopsies, reducing interobserver variability. Deep learning models distinguish between UC and Crohn’s Disease, while CADe systems accurately assess UC biopsies for prognostic prediction [5]. CNN models achieve high accuracy in assessing endoscopic and histological remission [6]. Precision medicine integrates domain insight with bioinformatics through machine learning, enhancing treatment strategies by predicting therapeutic responses to medications like infliximab [7]. Despite these advances, challenges arise in selection bias and the need for multi-centre data for AI algorithms’ effectiveness. AI-assisted colonoscopies require prospective studies for validation, and physician hesitancy highlights the importance of maintaining human involvement in diagnostic decision-making [8,9]. Financial incentives and payer requisites may foster the widespread adoption of AI-assisted diagnostics and treatments in clinical settings [10].

## 2. Methods

A targeted, non-systematic review of the literature relating to ulcerative colitis was undertaken. The PubMed and Scopus databases were searched to categorize a well-rounded understanding of the field of artificial intelligence and its developing role in the diagnosis and treatment of ulcerative colitis. Different combinations of keywords and phrases were used to determine information related to the title at hand. Some of the keywords included were ulcerative colitis, artificial intelligence, diagnosis, treatment, deep learning, machine learning, endoscopy, and neural networks. All materials published before 28 February 2024 were acceptable sources for this review. Only English articles that were published were included in this literature review.

## 3. An Overview of Ulcerative Colitis with Modern Diagnostic Approaches

Individuals with ulcerative colitis (UC) may experience the progression of proximal disease extensions, potentially advancing to pancolitis, along with structural and functional alterations, resulting in diminished quality of life and disability [11]. While ulcerative colitis may manifest mildly in some patients, it concurrently elevates the risk of cancer among afflicted individuals, with the extent and duration of the disease playing significant roles in determining this heightened susceptibility [12]. UC is quite more widespread than Crohn’s disease. Northern Europe and North America exhibit the highest incidence and prevalence rates of UC. Incidence ranges between 9 and 20 cases per 100,000 person-years, while prevalence ranges from 156 to 291 cases per 100,000 individuals. These values are increased in countries with developed industrial lifestyles. Within eastern countries and the southern hemisphere, the incidence of the disease is far lower, which implies that the interplay of environmental factors could play a crucial role in the initiation of UC [1]. Encompassing the patient’s history, physical exam, endoscopic evaluation, histopathological findings, radiographic changes and biochemical analysis is imperative for the diagnostic indications of the disease’s severity. Endoscopic evaluation serves as a critical tool in pinpointing the precise location and visualizing the extent of inflammation associated with ulcerative colitis, delineating nuances such as segmental or continuous patterns and varying degrees of severity. Moreover, it possesses the capability to discern non-inflammatory pathologies, including dysplasia, thereby contributing to comprehensive diagnostic insights. Advances in endoscopic techniques coupled with refined histological analyses hold the promise of enhancing with regard to the diagnostic and therapeutic outcome of ulcerative colitis. Advancements in endoscopic techniques and histological analysis hold the potential to improve outcomes in the identification and therapy of ulcerative colitis [13]. Indeed, the precision of endoscopic assessments hinges largely on the interpretation and biopsy obtaining experience of the performing endoscopist. While contemporary endoscopic devices commonly feature high-definition white light endoscopy (HD-WLE) with optical-enhancing chromoendoscopy capabilities, the primary challenge lies in the accurate interpretation rather than the visualization per se. A suboptimal quality endoscopy may result in delayed diagnosis and the onset of severe complications, underscoring the critical importance of both technical proficiency and interpretive acumen in endoscopic examinations for conditions such as ulcerative colitis [14]. Given that therapy decisions are impacted by endoscopic evaluation, inconsistencies among observers contribute to suboptimal patient outcomes. Disease severity scoring is semi-quantitative and is not frequently implemented. For instance, a study examining endoscopic scoring variability among 58 gastroenterologists demonstrated an interrater agreement of only 0.47 for Mayo endoscopic subscore ratings in UC patients and 0.33 for the Rutgeerts score in CD patients. This variability led the study authors to estimate that one-third of patients would undergo different management solely based on endoscopic findings [13].

## 4. The Scope of Artificial Intelligence and Its Subdivisional Networks

Artificial intelligence (AI) refers to computer algorithms capable of learning, problem solving and decision-making [15]. In the medical field, artificial intelligence spans across multiple areas such as gene sequencing, computational intelligence, intelligent diagnosis, and medical robotics. Within gastrointestinal endoscopy, AI technology has demonstrated superior performance compared to professional endoscopists in terms of accuracy for analyzing and processing vast patient data. This superiority has been a driving force behind the rapid adoption of AI in gastrointestinal endoscopy in recent years [16]. A key subdomain within AI is the artificial neural network (ANN), comprising input, hidden connections, and output layers. ANNs simulate the neural structure of the human brain, enabling decision-making through the weighted summation of evidence [17]. Machine learning (ML) represents another pivotal field within the domain of artificial intelligence. ML algorithms are designed to autonomously carry out tasks by deducing data patterns and overlaps without direct programming. This capacity allows machine learning to analyze extensive datasets, recognizing patterns to predict various disease attributes such as severity or prognosis. By leveraging ML techniques, healthcare professionals can gain valuable insights from complex medical data, thereby enhancing diagnostic accuracy, treatment planning, and patient care [15]. Deep learning is a subfield of ML. It is regarded as having many advantages in the clinical evaluation of UC patients. Recently, DL has significantly improved the image recognition ability of AI, thanks to the vast availability of data sets to provide learning materials for the neural networks. However, with the development of CNN, medical pattern recognition has made tremendous progress. Within the span of a few years, AI has made a remarkable breakthrough as a screening software for medical images in areas including, but not limited to, ophthalmology, pathology and neurology [18].

## 5. Artificial Intelligence in the Diagnosis of Ulcerative Colitis

In the realm of gastrointestinal endoscopy, AI is progressing along two primary avenues: the detection of mucosal lesions through computer-aided detection (CADe) and the characterization of these lesions via computer-aided diagnosis. Additionally, AI is employed in monitoring the endoscopic procedure itself. Although over 30 histological scoring systems have emerged for grading UC histology in recent years, their clinical utility remains constrained by their intricate nature. Even in clinical trials where these scores are utilized, there is significant interobserver variability, necessitating the use of expensive central reading systems staffed by highly qualified pathologists to mitigate discrepancies. Consequently, there is rapid growth in employing AI-based systems to automate the assessment of UC biopsies, aiming to standardize evaluations and reduce interobserver variability. Ongoing trials in this area show promising initial results. Initially, efforts concentrated on developing a Computer-Aided Detection (CADe) model to assess UC biopsies using eosinophil counts. While the system exhibited strong agreement with manual counts conducted by pathologists with the interclass correlation coefficient = 0.81–0.92, after thorough evaluation, it was found that there was no relationship between the number of eosinophils and total inflammatory activity. The IBD encompasses both Ulcerative Colitis and Crohn’s Disease, making their clinical diagnosis difficult to differentiate [5]. Park et al. developed a deep learning model aimed at distinguishing between the two diseases using RNA sequencing data derived from patients with inflammatory bowel disease (IBD) who had undergone endoscopy and had biopsy tissue samples recorded. By aligning the RNA sequence dataset to the human reference genome GRCh38 and quantifying the associated gene models, 19,596 protein-coding genes were included. The non-supervised learning algorithm revealed four entities to categorize the samples: UC normal, CD normal, UC inflammatory, and CD inflammatory. The supervised deep learning software was successfully able to classify inflammatory UC from inflammatory CD. Following the pruning process, robust classifiers for distinguishing between normal Crohn’s disease (CD) and normal ulcerative colitis (UC) were identified, which reinforced the ML abilities for RNA sequencing analysis of endoscopic mucosal tissue to differentiate between the two diseases [19].

Rio et al. mentioned researchers who focused on a simplified method focusing on assessing UC activity solely based on neutrophils, which represent the primary indicator of active inflammation. With the introduction of the PICaSSO histologic remission index, incorporated into a CADe system capable of accurately distinguishing histological activity from remission in UC biopsies [3]. Iaucci et al. followed up on the PICaSSO index and used CADe systems to analyze UC biopsies for prognostic prediction. With 535 digitalized biopsies graded according to the PHRI, the system’s ability to differentiate between histological activity and remission scored a sensitivity of 89% and a specificity of 85%. And an overall AUROC of 0.97, maintaining a promising diagnostic performance despite a mixture of severities in grades [20]. Furthermore, the incorporation of AI in endoscopic procedures would help improve the exactness and accuracy of evaluating disease severity, as well as eliminating human factors for subjectivity, bias, and variability. Gutierrez et al. developed and trained an artificial neural system to evaluate the Endoscopic Mayo Score (EMS) using colonoscopies obtained from Etrolizumab trials. The outcomes exhibited significant promise, with AUROC values of 0.84, 0.85, and 0.85 for Mayo Clinic Endoscopic Subscores ≥1, ≥2, and ≥3, respectively [21]. Jiang et al., who utilized CNN models for the objective analysis of endoscopic disease activity and UC predictive remission, provided excellent data to support the assessment of inflammation in UC patients undergoing endoscopies to predict histological remission. The MES-CNN model attained a diagnostic accuracy of 97.04% for assessing endoscopic remission of UC and an accuracy of 90.15% in severity evaluation. In predicting histological remission, the CNN models achieved an accuracy of 91.28% and a kappa value of 0.826, surpassing the accuracy of endoscopists of 87.69% [6]. With endoscopes being utilized in diagnostic mucosal healing, Huang underscored the reliability of the combination between CADe and endoscopic diagnostics by achieving 94.5% accuracy for diagnostic mucosal healings with the computer-aided diagnostic system using deep learning and machine learning to classify mucosal healings [22].

Other indexes have been utilized in the quantitative assessment of the histopathological findings in UC. Researchers used a random forest classifier built on 13 human interpretable features exported from cell and tissue models, which were used as accurate prediction markers for the Nancy Histological Index scores, which yielded a weighted kappa of 0.91 and a spearman correlation of 0.89. The absence of neutrophil extravasation was utilized as a prediction marker for histological remission, which achieved an accuracy of 0.97, indicating the implications and power of computer assessments of UC histopathology [23]. A separate study carried out by Bhambhavi et al. successfully trained a Convolutional Neural Network (CNN) model through static images captured during endoscopy to identify and categorize images based on the endoscopic Mayo score (EMS). Their final model demonstrated robust performance, achieving an AUC of 0.89 for MES 1 disease, 0.86 for MES 2 disease and 0.96 for MES 3 disease. The overall accuracy of the model reached 77.2% [24]. The rapid integration of AI gives the opportunity to operate with a large database to detect occult disease patterns [4]. A study conducted by Chen et al. collected data from 187 patients with ulcerative colitis (UC), which demonstrated the significant advantages of CAD systems in medical imaging. This study revealed that CAD systems not only enhance precision and sensitivity but also outperform traditional imaging methods by providing more detailed imaging information, automating processes, and ensuring better reproducibility. The study utilized data from 187 patients, with 525 validation sets collected from 100 patients. Additionally, with 12,900 endoscopic images collected from the final 87 patients, the information was utilized to train the CAD system. Each endoscopic image was annotated with its corresponding histological diagnosis. The CAD system exhibited substantial promise, achieving a diagnostic sensitivity of 74%, a specificity of 97% and an overall accuracy of 91% in identifying histological inflammation related to ulcerative colitis. These results underscore the potential of CAD systems to fully automate the identification of UC-related histological inflammation, thereby enhancing diagnostic capabilities and patient care [17]. Presents the usage of CAD by different institutions [25]. Another aspect of AI is the deep neural network (DNN). According to much research, deep neural networks can evaluate the remission and activity of UC with only endoscopic analysis, excluding the necessity of biopsies. It is believed that the objectivity, coherence, and accuracy of these systems are like those of a professional endoscopist [16]. Sutton et al. conducted a comparative analysis of various convolutional neural network (CNN) architectures subjected to a varied set of 8000 labelled endoscopic images sourced from HyperKvasir. HyperKvasir stands as the most extensive video and image dataset available for gastrointestinal tract conditions to date. The dataset demonstrated robust capabilities in accurately discerning between UC and non-UC pathologies, thereby aiding in determining the Mayo score of endoscopic disease severity. The models were initialized with ImageNet weights, and hyperparameters were optimized through Grid Search employing fivefold cross-validation. The DenseNet121 architecture emerged as the most effective, reaching an accuracy of 87.50% and an AUC of 0.90, surpassing the baseline prediction metrics of 72.02% accuracy and 0.50 AUC obtained by predicting the majority class [26]. 

Different papers exploring the auto-grading of the endoscopic modality have also highlighted the benefits of employing deep learning models. For example, Takenaka et al. conducted an evaluation using a DL network-based model on endoscopic imaging of patients suffering from UC. Their model demonstrated high accuracy, reaching 90.1% when assessing endoscopic imaging from 40,758 UC patients with signs of remission from the endoscopy, all of whom had a UC Endoscopic Severity Index Score of 0. Alongside this, Yao et al. introduced modifications to the video operation model by segmenting endoscopic videos into 1 FPS imaging stacks. They then automated processes such as rotation, fragmentation and pre-processing of the images to ensure conformity to a standard scale. The resultant model successfully generated Mayo endoscopic subscores for patients in an automated manner [16]. Endoscopic and histological remission (ER and HR) are therapeutic goals in UC. To improve histological prediction, Iacucci et al. developed a convolutional neural network to separate ER/activity from histology prediction and white light endoscopy flare risk. The CNN system demonstrated a sensitivity of 72% and a specificity of 87%, with an AUROC of 0.85 for detecting ER (UCEIS ≤ 1) in white light endoscopy videos. In virtual chromoendoscopy videos (PICaSSO ≤ 3), the sensitivity was 79%, the specificity was 95%, and the AUROC was 0.94 for ER detection. By incorporating PICaSSO, the CNN could provide a thorough histologic, clinical, and endoscopic assessment [27]. Hamamoto et al. utilized the no-code AI software Version 1/2017 “Teachable Machine” to develop a model capable of recognizing patterned individual differences between histological images of ulcerative colitis, adenocarcinoma, non-UC coloproctitis, and control samples. They curated a dataset consisting of 5100 histological images, which were designed to train the software, and a further 900 for testing, all meticulously annotated by pathologists. Their model achieved remarkable accuracies of 0.99 for UC, 1.00 for non-UC coloproctitis, 0.99 for adenocarcinoma, and 0.99 for control samples. This study demonstrated the efficacy of a user-friendly, no-code AI platform in accurately identifying the distinct histologic patterns associated with UC [28].

Biomarker prediction has been explored using least absolute shrinkage and selection operators (LASSO) and random forest (RF). By using LASSO and RF to screen signature genes through a GEO database, the ANN and ROC curves were used to judge the diagnostic significance of these tools regarding signature genes. The intersection of LASSO, RF, and WGCNA analyses revealed eight signature genes: DUOX2, MMP10, SLC6A14, S100A8, GREM1, CXCL1, IL-1B and TCN1. These markers are seen as credible for the diagnosis of UC due to their role in immune mediation for the advancement of the disease [29]. In a separate study, Wang et al. focused on identifying potential biomarkers for ulcerative colitis (UC) and understanding their association with immune infiltration. By merging two datasets, the researchers analyzed 193 UC samples and 42 normal samples. Using computational methods, 102 differentially expressed genes (DEGs) were discovered between UC and normal samples, with pathways related to interleukin-17 and cytokine receptors being significantly enriched. Through machine learning techniques and ROC analysis, five genes (DUOX2, DMBT1, CYP2B7P, PITX2, and DEFB1) were identified as promising diagnostic markers for UC. Furthermore, analysis of immune cell infiltration revealed correlations between these biomarkers and various immune cells, such as regulatory T cells, CD8 T cells, and macrophages. These findings suggest that these biomarkers may provide valuable insights into the progression of UC and its underlying immune mechanisms [30]. Li et al. underscored the potential for biomarker usage in the screening diagnostics for ulcerative cancer by selecting patients with mucosal intestinal biopsy of UC from the GEO database. By employing machine learning and WGCNA analysis, potential UC biomarkers were isolated with ROC curves employed for result validation, while the mechanisms of these marker genes were predicted through immune cell infiltration analysis, co-expression analysis, and competitive endogenous network analysis. The study identified five potential biomarkers, TIMP1, IRAK3, HMGCS2, APOBEC3B and SLC6A14, with diagnostic and therapeutic relevance for UC. It verified their involvement in UC occurrence and progression through immune infiltration analysis and proposed a plausible RNA regulatory pathway governing UC advancement [31]. Khorsani et al. utilized an interplay between a developed feature selection algorithm and a support vector machine classifier to create a model that could differentiate between diseased and healthy individuals through the expression values of 32 genes in colon samples. The model accurately detected all active cases and sustained an average precision of 0.62 for inactive cases. The final UC detection model demonstrated superior performance compared to a biomarker discovery software using machine learning, BioDiscML, in terms of average precision [32]. An individual study aimed to develop an effective diagnostic model for ulcerative colitis (UC). By analyzing microarray data from GSE48634 and GSE87473 obtained from GEO, 126 differentially expressed genes (DEGs) between UC patients and normal samples were discovered. GO and KEGG analyses suggested enrichment in immune-related processes and pathways. Immune cell infiltration analysis showed significant differences between UC patients and normal. A logistic regression model incorporating expression levels of selected genes achieved an average AUC of 0.8497 in the training set (GSE87473) and 0.7208 in an independent validation set (GSE48634). Notably, hub genes like DEFA5, REG1A, REG1B, DEFA6 and REG3A were identified as potentially associated with UC progression. Overall, the results indicate that this five-gene logistic regression model holds promise for reliable UC diagnosis [33].

## 6. Artificial Intelligence in the Treatment of Ulcerative Colitis

With the development of AI, there has been a notable shift in the perception of the treatment goals for ulcerative colitis. In the evolution of therapeutic strategies, the traditional goal of attaining clinical remission has undergone a significant shift with the emergence of musical healing (MH). This paradigm shift reflects advancements in medical therapeutics and aims to redefine treatment endpoints capable of fundamentally altering the natural course of disease. MH is associated with a reduced risk of complications, including neoplasia, hospitalization, surgery and relapse. Despite the multitude of endoscopic scoring systems proposed in recent years, a consensus regarding the definition of MH remains elusive [4]. According to the International Organization for the Study of Inflammatory Bowel Disease (IOIBD), MH can be identified as an absence of ulcers and friability in every accessible segment of the colonic mucosa [34]. A report by Colombel et al. [35], In numerous studies, MH has typically been characterized as a Mayo Endoscopic Score (MES) of 0–1, irrespective of histological findings. Major clinical trials have established endoscopic remission as MES ≤ 1. Nonetheless, while clinical symptoms have been the primary focus in most studies on UC, endoscopic remission has commonly been incorporated as a secondary endpoint [14,15]. 

In 2020, Hota et al. conducted a comprehensive investigation into the perioperative and treatment outcomes associated with robotic, laparoscopic and open surgeries in the management of Crohn’s disease (CD). Their study involved the analysis of a database comprising 5158 patients diagnosed with CD. Leveraging Convolutional Point Transformer (CPT) codes, they meticulously identified the surgical procedures employed for patient bowel resection. Comparative analyses across the three surgical modalities were conducted, with a particular focus on assessing the incidence of anastomotic fistula. Employing sophisticated multivariate analysis techniques, the researchers derived a confidence interval for the dominance ratio of 95%. Notably, their findings indicated that robotic surgery demonstrated non-inferiority as a treatment option for bowel resection in ulcerative colitis recipients. This research sheds valuable light on the differences in surgical approaches and their associated outcomes, contributing to the refinement of treatment strategies in UC management [36]. Given the intricate nature of inflammatory bowel disease (IBD) pathogenesis, relying solely on the interpretation of individual omics data sets often proves inadequate for understanding complex biological processes. Hence, it is crucial to perceive the previously discussed omics methodologies as a unified entity. The fusion of several omics techniques into a network presents potential for elucidating the pathways implicated in pathogenesis and facilitating the discovery of different subgroups, thereby enhancing therapy regimens for IBD. Conducting simultaneous assessments of diverse molecules spanning transcriptomic, genomic, microbiome, proteomic and metabolomic levels is feasible, with subsequent integration of findings into multiomics models. These biomarkers enable a deeper understanding of disease pathogenesis, facilitate the discovery of promising predictive biomarkers, and promote the development of early patient-tailored treatment plans. Several ongoing multiomics projects focus on investigating IBD heterogeneity to enhance precision management strategies [37]. The treatment goal for IBD has shifted from a typical clinical remission to a more comprehensive objective of achieving deep remission or mucosal healing. As a result, clinical decision-making has become increasingly challenging for both patients and clinicians. Presently, the efficacy and anticipated tolerability of novel treatment options typically guide clinical decisions. However, numerous challenges persist in optimizing treatment strategies, enhancing the long-term prognosis, and altering the natural course of IBD. Machine learning (ML) offers promise due to its capacity to rapidly compile and apply medical information and imaging modalities to generate therapeutic outcomes, enabling predictions regarding IBD progression or the effectiveness of specific medications [17].

Liu et al. demonstrated a new outlook for combining digital histopathological histomic features and ML algorithms to predict the response of paediatric patients to certain therapies. This will aid in patient identification and classification into those who can achieve remission without the use of steroids when on mesalamine monotherapy. The algorithm was trained on 187,571 informative patches from rectal biopsies stained with H-E. A total of 292 samples from treatment-naive paediatric patients with ulcerative colitis were used within the multicenter inception cohort study. With the machine learning algorithm being initially trained on 250 histomic features, it achieved an AUROC of 0.87 with a 0.90 accuracy within the WSI level for treatment response prediction. The potential for the algorithm’s clinical usability was reinforced when a subdivision of 18 histomic features demonstrated a similar performance, achieving 0.89 and 0.90 for the AUROC and accuracy, respectively, highlighting its potential for a standardized clinical use case within practical boundaries [38,39].

With infliximab being a first-line immunomodulator therapy for ulcerative colitis, it is imperative to assess patients who show primary non-responsiveness to the drug [40,41]. This affects around one-third of patients [42]. A study by Chen et al. assessed the GEO datasets and utilized the RobustRankAggreg software to characterize and discover differentially expressed genes between primary non-responders and those who can benefit from the drug. For selected genes to obtain a predictive value, ANN was employed, with primary results suggesting that the interplay between CHP2, CDX2, NOX4, RANK, HSD11B2 and VDR provides a satisfactory indicator of a personalized therapeutic response to infliximab. The repeated overall (AUC) ranged from 0.850 to 0.103. Alongside this, the utilization of an independent GEO dataset confirmed the predictive value of the six differentially expressed genes for primary non-responsive patients to infliximab, yielding an overall AUC range of 0.759 ± 0.065. Given that the detection of proteins does not necessitate that fresh tissue and can circumvent multi-biopsies, the researchers analyzed key information for protein detection to measure the suitability of RNA level by employing immunohistochemistry staining of biopsy tissues obtained from the colon of UC patients treated with infliximab, alongside ROC analysis, to delve deeper into the clinical application potential of the six DEGs at the protein level. RANK and VDR were confirmed to be associated with infliximab efficacy through immunohistochemistry of the colonic tissue. This provides a bridge between the impact of ANN software Chainer v 7.8.1 on the prediction of patient responses, thus allowing a more tailored approach for patients with selected genes [7]. A similar study carried out by Popa et al. underscores the ability of artificial intelligence to be utilized for personalized therapy management, having attained outstanding performance in predicting disease activity at one year, with a test set accuracy of 90% and an AUC of 0.92, along with a validation set accuracy of 100% and an AUC of 1. The proposed machine learning solution demonstrates potential as a valuable tool for aiding clinicians’ decisions. Upon validation across independent, external cohorts of patients, it could support clinical judgments regarding dose adjustments or the transition to alternative biological therapeutics [43]. The role of machine learning has been emphasized with an array of therapeutics, with biologics such as vedolizumab being hypothetically optimized for personal therapeutic use in patient treatment, with researchers using the algorithm to identify patients who would not achieve steroid-free clinical remission at week 22; therefore, allowing a new clinical approach to be curated with alternative biologics that would potentially improve their therapeutic journey [44]. With the development of precision medicine, its clinical applications allow patients to be treated based on their disease and reaction to drug administration [45]. Gardiner et al. wanted to combine domain insight with bioinformatics through a machine learning mechanism that would assist in predicting the changes and variability in drug responses between different patients. Using the ML software RandomForest RF (version 4.7-1.1); xgboost 2.0.3 as a building block, these researchers used the data produced by the ML to interpret the response of patient fresh tissue receiving pharmaceuticals under preclinical testing. As a means of assessing the effectiveness of drugs, they evaluated the decrease in the release of the inflammatory cytokine TNFα from fresh IBD tissues with and without the presence of test drugs. Initially, they investigated the impact of a mitogen-activated protein kinase (MAPK) inhibitor, but later demonstrated that this methodology could be extended to examine other targets, test drugs, or mechanisms of interest. Given patient variations ranging from age to gender or condition, their findings revealed differences in drug efficacy when assessed through ex vivo assays. Furthermore, they correlated new genetic polymorphisms with variations in patient responses to the anti-inflammatory treatment BIRB796 (Doramapimod). This allowed their approach to not only model drug responses in IBD but also identify the most influential features, employing a transparent machine learning precision medicine strategy [46].

## 7. The Post-Operative Role of Artificial Intelligence

Patients with ulcerative colitis have a high risk of postoperative complications following a total abdominal colectomy [47]. By utilizing machine learning, researchers were able to predict the rate of minor postoperative complications in patients with high-risk ulcerative colitis. Data from 32 patients were used for the statistical analysis, focused on preoperative therapy, biographical data, blood chemistry, nutritional status, surgical technique and blood transfusion. The algorithm demonstrated that low preoperative serum albumin levels were associated with a higher risk of minor infection, and if the length of the preoperative stay was >4 days, the body temperature was >37.5 °C, and the blood transfusion massed to 1 or more units, the risk of infectious morbidity rose significantly. Despite a small sample size, its use case can be applied to a suitable clinical scenario, given that a larger dataset will be available for the learning algorithm to be trained on [48]. Mizuno explored the predictive capabilities of artificial intelligence and deep learning to predict pouchitis in patients with ulcerative colitis following ileal pouch-anal anastomosis. In this paper, a total of 43 patients were included, with pouchitis occurring in 33% of patients after ileostomy closure. The CNN model measured predictive rates of pouchitis following ileostomy closure through fivefold cross-validation. Results suggest that most patients’ modified pouchitis disease activity index scores, which did not match before and after the ileostomy closure, had worse scores than before. The predictive rate of pouchitis using the deep learning model was 20% greater than that of using the modified pouchitis disease activity index. This was highlighted, where the predictive accuracy for pouchitis determined by the area under the curve using the DL model was 84%. In contrast, the predictive accuracy for pouchitis using the modified pouchitis disease activity index prior to ileostomy closure was 62%, suggesting that the DL program could be used to determine early interventions for pouchitis [49].

We included a table to demonstrate and summarize the use of artificial intelligence and how it is being integrated into UC diagnostics and therapeutics (Table 1).

## 8. Prematurity in the Advancement and Utilization of Artificial Intelligence in Ulcerative Colitis

With the expansive and instrumental development in the field of medical artificial intelligence, the neural software’s, capabilities suffer from some drawbacks that impair their utilization in modern medicine. It is essential to acknowledge and address the inherent challenges and limitations. These include issues like selection bias, spectrum bias and the simplistic nature of algorithm development, which may lead to inappropriate generalizations of results. The current evidence is primarily based on retrospective data used to train AI algorithms. However, these datasets, often derived from clinical trials of investigational drugs, may not fully represent real-world scenarios. Moreover, CNN models trained on data from single centres have shown limited performance in broader applications, highlighting the importance of acquiring multicenter data and externally validating AI algorithms. Prospective studies, aligned with the new CONSORT-AI and SPIRIT guidelines, are crucial to assessing the effectiveness of AI in managing inflammatory bowel disease (IBD). Challenges also exist in AI-assisted colonoscopies, particularly in adapting algorithms from still images to real-life video colonoscopies. Analyzing raw full-motion videos and distinguishing informative frames (e.g., affected mucosa) from non-informative ones poses a significant challenge. Real-time analysis is necessary to provide insights into disease type, severity, treatment response and neoplasia development [4].

In a survey encompassing 487 pathologists across 59 nations, the predominant sentiments included concerns regarding physician distrust, technophobia, liability, and the fear of potential replacement by AI, all of which may contribute to hesitancy in adopting AI tools. Most respondents (72.0%) expressed optimism about the potential positive impact of AI on diagnostic efficiency. However, most respondents also emphasized the importance of maintaining human involvement in the diagnostic decision-making process [8,9]. Within the existing fee-for-service reimbursement structure, integrating AI may pose challenges; nevertheless, in a value-based model prioritizing enhanced quality and reduced costs, AI is poised to serve as a valuable supplement. It is conceivable that payers might stipulate coverage for new drugs or treatment continuations contingent upon the availability of accurate assessments of patients’ disease activity facilitated by AI-driven technology. Consequently, payer requisites for reimbursement are anticipated to be pivotal in fostering the uptake of AI within healthcare. Notably, the widespread adoption of AI-assisted optical biopsy in clinical settings may hinge on the provision of financial incentives through reimbursement fee codes [10].

The diversity among data sources utilized for training and validation, such as the presence of missing or irrelevant data, can diminish the performance of AI models when applied in real-world scenarios. Furthermore, the intricate nature of real-world conditions may not be sufficiently integrated into AI algorithms, potentially resulting in lower accuracies of AI tools in practical settings compared to what is reported in the academic literature [8]. AI models may face challenges with rare clinical scenarios due to their limited representation in training datasets. Overcoming this limitation requires the availability of high-quality, standardized datasets that encompass geographic, technical and patient diversity [50].

## 9. Discussion

Ulcerative colitis (UC) imposes a significant burden on affected individuals, with a spectrum of disease severity ranging from mild manifestations to potentially life-altering complications. Epidemiological data underscore the global impact of UC, with incidence rates ranging from 9 to 20 cases per 100,000 person-years and prevalence rates between 156 and 291 cases per 100,000 individuals. Notably, regions with developed industrial lifestyles exhibit higher prevalence rates, implicating environmental factors in disease initiation [1].

Endoscopic evaluation stands as a cornerstone in UC diagnosis, offering critical insights into disease localization and severity. However, challenges persist in achieving consistent interpretations of endoscopic findings. For instance, interrater agreement for Mayo endoscopic subscore ratings among gastroenterologists remains modest, with reported values of 0.47 [13]. These inconsistencies underscore the need for standardized diagnostic approaches. Artificial intelligence (AI) holds promise for revolutionizing UC diagnosis and management. Advanced AI-driven technologies, such as computer-aided detection (CADe) and computer-aided diagnosis, aim to mitigate interobserver variability in assessing UC biopsies. Notably, CADe models using deep learning techniques exhibit strong agreement with manual eosinophil counts conducted by pathologists, with an interclass correlation coefficient ranging from 0.81 to 0.92 [5].

The evolution of treatment goals for ulcerative colitis (UC) has been profoundly influenced by advancements in artificial intelligence (AI), leading to a paradigm shift in therapeutic strategies. Traditionally, the primary goal has been to achieve clinical remission, but the emergence of musical healing (MH) has introduced a new perspective. MH, associated with a reduced risk of complications such as neoplasia, hospitalization, surgery, and relapse, aims to redefine treatment endpoints to fundamentally alter the natural course of the disease. Despite the proliferation of endoscopic scoring systems, a consensus on the definition of MH remains elusive. However, according to the International Organization for the Study of Inflammatory Bowel Disease (IOIBD), MH can be identified as the absence of ulcers and friability in every accessible segment of the colonic mucosa. Major clinical trials have established endoscopic remission as a Mayo Endoscopic Score (MES) of 0–1, regardless of histological findings [34,35]. In 2020, Hota et al. conducted a comprehensive investigation into perioperative and treatment outcomes associated with robotic, laparoscopic, and open surgeries in managing Crohn’s disease (CD). Analyzing a database of 5158 CD patients, they identified surgical procedures using Convolutional Point Transformer (CPT) codes. Their findings indicated that robotic surgery demonstrated non-inferiority as a treatment option for bowel resection in UC recipients. The intricate nature of inflammatory bowel disease (IBD) pathogenesis necessitates a holistic approach [36]. Assessing primary non-responsiveness to infliximab, a first-line immunomodulator therapy for UC, is crucial. Chen et al. identified genes like CHP2, CDX2, NOX4, RANK, HSD11B2 and VDR as predictive markers for the therapeutic response to infliximab. Immunohistochemistry confirmed the associations of RANK and VDR with infliximab efficacy at the protein level [7]. In summary, the integration of AI, multiomics approaches, and machine learning algorithms is revolutionizing the management of UC. These advancements offer personalized treatment strategies, improve treatment outcomes, and enhance our understanding of disease pathogenesis.

Machine learning algorithms offer valuable predictive insights into postoperative outcomes. For instance, algorithms predict minor postoperative complications based on a range of factors, including preoperative serum albumin levels, length of preoperative stay, and perioperative variables [48]. These predictive models enable proactive management strategies to mitigate postoperative risks and enhance patient recovery. Incorporating AI-driven approaches into UC management represents a paradigm shift, offering opportunities to enhance diagnostic accuracy, optimize treatment strategies, and improve postoperative outcomes. However, realizing the full potential of AI in clinical practice necessitates further research, validation, and integration into existing healthcare frameworks, ensuring equitable access and improved patient-centred care.

## 10. Conclusions

When considering the potential of artificial intelligence, its ability to enhance patient care from a diagnostic and therapeutic perspective shows signs of promise. With the integration of neural networks and convolutional layers, the capabilities of this intelligent software allow for expansive cohort analysis and prediction, giving physicians a more defined understanding of how to approach each individual patient.

For the true utilization of artificial intelligence, some roadblocks must be addressed. The datasets available to AI may not truly reflect the real-world, which would prevent its impact in all clinical scenarios when dealing with a spectrum of patients with different backgrounds and presenting factors. Moreover, there is still a sense of physician distrust and technophobia, sparking a certain fear factor when entrusting the software with the life of a patient. This leads to physicians maintaining their stance of being the single driving factor for the management of a patient in terms of their diagnostic and therapeutic plan.

Considering this, the shift in medical diagnostics and therapeutics is coinciding with evolving technology. With a continuous advancement in artificial intelligence programming and a perpetual surge in patient datasets, these networks can be further enhanced and supplemented with a greater cohort, enabling better outcomes and prediction models for the future of modern medicine.

## Figures and Tables

**Table 1:** Performance results of existing studies.

Study	Publication Year	Diagnostics or Therapeutics	Dataset Utilized	AI Model	Results	Conclusion
Becker et al. [21]	2021	Diagnostic	1672 endoscopic videos	End-to-end computer-assisted diagnosis system based on deep learning	AUC = 0.84 for Mayo Clinic Endoscopic Subscore ≥1, 0.85 for ≥2, and 0.85 for ≥3), with reduced manual annotation required.	The evaluation on 1672 endoscopic videos from the etrolizumab Phase II Eucalyptus and Phase III Hickory and Laurel trials demonstrates high accuracy and robustness, which provides an increase in efficiency and standardization in the diagnosis of UC within a clinical setting.
Bhambhvani HP et al. [24]	2021	Diagnostics	777 Still images of endoscopies	101-layer convolutional neural network model	The model achieved AUCs of 0.96 for MES 3, 0.86 for MES 2, and 0.89 for MES 1 classifications, with an overall accuracy of 77.2%. Across the MES categories, it showed an average specificity of 85.7%, a sensitivity of 72.4%, a PPV of 77.7%, and an NPV of 87.0%.	They have illustrated the robust capability of a deep learning model to effectively classify different grades of endoscopic disease severity in ulcerative colitis patients.
Sutton et al. [26]	2022	Diagnostics	8000 labelled endoscopic still images derived from HyperKvasir	InceptionV3 ResNet50 VGG19 DenseNet121	The DenseNet121 architecture achieved the highest accuracy (87.50%) and Area Under the Curve (AUC) (0.90), surpassing the majority class prediction (‘no skill’ model), which attained 72.02% accuracy and 0.50 AUC.	They achieved moderate-to-good performance, distinguishing between mild and moderate-to-severe ulcerative colitis (UC), using a relatively small public dataset of endoscopy images. This achievement is notable, considering that images with global labels, such as Mayo endoscopic subscores, typically necessitate larger datasets to achieve satisfactory performance.
Iacucci et al. [20]	2023	Diagnostic	535 digitalized biopsies	VGG16 CNN	The system effectively distinguished between histological activity and remission, achieving sensitivities and specificities of 89% and 85% (PHRI), 94% and 76% (Robarts Histological Index), and 89% and 79% (Nancy Histological Index), respectively. Moreover, it accurately predicted endoscopic remission/activity with 79% and 82% accuracy for UC endoscopic index of severity and Paddington International virtual ChromoendoScopy ScOre, respectively. The hazard ratios for disease flare-up between histological activity and remission groups were 3.56 for PHRI assessed by pathologists and 4.64 for AI-assessed PHRI.	The CAD system accurately differentiated between disease remission and activity, as defined by the PHRI, RHI, and NHI, in real-time. Additionally, it effectively predicted corresponding endoscopic activity and assessed the risk of flare-up.
Iacucci et al. [27]	2023	Diagnostic	1090 endoscopic videos	ResNet50 CNN	The AI system detected endoscopic remission (UCEIS ≤ 1) in WLE videos with 72% sensitivity, 87% specificity, and an AUROC of 0.85. For VCE videos (PICaSSO ≤ 3), sensitivity was 79%, specificity 95%, and AUROC 0.94.	The system effectively differentiated between endoscopic remission/activity and predicted HR and clinical outcomes from colonoscopy videos. It represents the inaugural computer model designed to detect inflammation/healing on VCE using PICaSSO and the pioneering computer tool offering comprehensive clinical, endoscopic and histologic assessments.
Yang et al. [29]	2023	Diagnostics	The gene expression profiles were obtained from the GEO database and subsequently received preprocessing and normalization using R software.	LASSO Random Forest	Through the intersection of LASSO, RF, and WGCNA results, 8 signature genes were discerned: TCN1, S100A8, DUOX2, CXCL1, IL-1B, SLC6A14, GREM1, and MMP10.	Credible potential biomarkers for the diagnosis and therapy of UC were identified through the discovery of 8 signature genes. These biomarkers are integral to the immune response underlying the onset and progression of UC, facilitated by reciprocal interactions between the signature biomarkers and immune-infiltrated cells.
Wang et al. [30]	2023	Diagnostic	Two datasets were merged to obtain 193 UC samples and 42 normal samples.	LASSO SVM-RFE	In our analysis, 102 differentially expressed genes (DEGs) were identified, with 64 showing significant upregulation and 38 exhibiting significant downregulation. Machine learning methods and ROC tests validated DUOX2, DMBT1, CYP2B7P, PITX2, and DEFB1 as pivotal diagnostic genes for UC.	Prospective biomarkers for UC, including DUOX2, DMBT1, CYP2B7P, PITX2, and DEFB1, were identified. These biomarkers, along with their associations with immune cell infiltration, may offer a novel perspective on understanding the progression of UC.
Li et al. [31]	2022	Diagnostic	The GEO database was used to obtain gene data sets.	LASSO SVM-RFE	A total of 107 differentially expressed genes were identified, predominantly linked to biological functions like humoral immune response and inflammatory response. From this set, five marker genes were meticulously screened, revealing associations with M0 macrophages, quiescent mast cells, M2 macrophages, and activated NK cells in terms of immune cell infiltration.	The study identified five biomarkers—IRAK3, HMGCS2, APOBEC3B, SLC6A14, and TIMP1—as potential aids in the diagnosis and treatment of UC. It verified their involvement in the onset and advancement of UC through immune infiltration analysis and proposed a potential RNA regulatory pathway governing UC progression.
Khorasani et al. [32]	2020	Diagnostic	The NCBI Gene Expression Omnibus database (GEO) was utilised for expression profiling studies using colonic samples from UC subjects	SVM DRPT	Achieving flawless detection of all active cases, the model exhibited an average precision of 0.62 in identifying inactive cases. This performance was benchmarked against results from prior studies and a machine learning-based biomarker discovery tool, BioDiscML, recently introduced into the scientific field.	In terms of average precision, the final model for detecting UC demonstrates superior performance.
Jiang et a [6]	2023	Diagnostics	12,257 endoscopic images	Inception-ResNet-v2	The MES-CNN model attained an accuracy of 97.04% in diagnosing endoscopic remission in UC cases. Additionally, the MES-CNN and UCEIS-CNN models demonstrated accuracies of 90.15% and 85.29%, respectively, in evaluating the endoscopic severity of UC. In predicting histological remission, CNN models achieved accuracy and kappa values of 91.28% and 0.826, respectively, surpassing the accuracy achieved by human endoscopists (87.69%).	Based on evaluations of MES and UCEIS by expert gastroenterologists, the proposed artificial intelligence model provides accurate assessments of inflammation in UC endoscopic images. Furthermore, it demonstrates reliable predictive capability for histological remission
Chen et al. [7]	2021	Therapeutics	Independent GEO dataset.	ANN	The study suggests that a combination of six genes—CDX2, CHP2, HSD11B2, RANK, NOX4, and VDR—accurately predicts patients’ response to IFX therapy, with a repeated overall AUC ranging from 0.850 ± 0.103. The validation using an independent GEO dataset confirms the predictive value of these genes, with an overall AUC range of 0.759 ± 0.065 for forecasting patient non-response (PNR) to IFX.	The study established a correlation between RNA and protein models, with both being accessible. However, the composite signature of VDR and RANK proves more favourable for clinical application. This composite signature could potentially guide the pre-selection of patients likely to benefit from pharmacological treatment in the future.
Popa et al. [43]	2020	Therapeutics	55 UC Patients	Multi-layered Perceptron Neural Network Model	The classifier demonstrated outstanding performance in predicting disease activity at one year. On the test set, it achieved an accuracy of 90% and an AUC of 0.92. Meanwhile, on the validation set, it attained a perfect accuracy of 100% and an AUC of 1.	After validation on independent external patient cohorts, the ML solution could serve as a valuable tool for clinicians, aiding them in decisions regarding dosage adjustments or transitions to alternative biologic agents.
Gardiner et al. [46]	2022	Therapeutics	25 patient organoculture assay data sets	RF XGBoost SVM KNN Adaboost	The top-performing model accurately predicted TNFα levels using demographic, medicinal and genomic features, achieving a remarkably low error rate of only 4.98% on unseen patients. Additionally, the findings revealed differences in drug effectiveness, as measured by ex vivo assays, among patients based on gender, age or condition. Moreover, new genetic polymorphisms were identified, highlighting their role in influencing variations in patient response to the anti-inflammatory treatment BIRB796 (Doramapimod).	They showcased the promise of merging preclinical functional assessments of drug effectiveness and inter-patient variability in drug response. By integrating cutting-edge omics, bioinformatics and ML/AI methodologies, they introduced a novel approach to crafting precision medicine strategies during the initial phases of drug development.
Miyoshi et al. [44]	2021	Therapeutics	34 Patients	RF	During validation with external Cohort 2, the prediction model exhibited positive and negative predictive values of 54.5% and 92.3%, respectively. This tool proved valuable in identifying UC patients unlikely to achieve SFCR at week 22 while undergoing VDZ therapy.	This study demonstrates the feasibility of personalized treatment for UC through machine learning with real-world data, serving as a proof-of-concept.
